# High-Resolution Mass Spectrometry for In Vivo Proteome Dynamics using Heavy Water Metabolic Labeling

**DOI:** 10.3390/ijms21217821

**Published:** 2020-10-22

**Authors:** Rovshan G. Sadygov

**Affiliations:** Department of Biochemistry and Molecular Biology, The University of Texas Medical Branch, Galveston, TX 77555, USA; rgsadygo@utmb.edu

**Keywords:** protein turnover, LC–MS, rate constant, high resolution MS

## Abstract

Cellular proteins are continuously degraded and synthesized. The turnover of proteins is essential to many cellular functions. Combined with metabolic labeling using stable isotopes, LC–MS estimates proteome dynamics in high-throughput and on a large scale. Modern mass spectrometers allow a range of instrumental settings to optimize experimental output for specific research goals. One such setting which affects the results for dynamic proteome studies is the mass resolution. The resolution is vital for distinguishing target species from co-eluting contaminants with close mass-to-charge ratios. However, for estimations of proteome dynamics from metabolic labeling with stable isotopes, the spectral accuracy is highly important. Studies examining the effects of increased mass resolutions (in modern mass spectrometers) on the proteome turnover output and accuracy have been lacking. Here, we use a publicly available heavy water labeling and mass spectral data sets of murine serum proteome (acquired on Orbitrap Fusion and Agilent 6530 QToF) to analyze the effect of mass resolution of the Orbitrap mass analyzer on the proteome dynamics estimation. Increased mass resolution affected the spectral accuracy and the number acquired tandem mass spectra.

## 1. Introduction

The use of metabolic labeling with heavy water for in vivo studies of protein turnover was first described [1] in 1941. Since then, heavy water has been used to label various experimental model species such as fish [2], rats [3], and mice [4]. At low doses of exposure, no adverse effects have been observed in humans who have continuously consumed ^2^H_2_O-enriched water for several months [5,6]. Using the time course of deuterium incorporation, the proteome dynamics of various tissues such as liver [4,7,8], heart [3,9], kidney [9], and muscle [10,11], as well as blood plasma [12,13], have been studied. 

Advances in mass spectrometry (MS) and separation techniques have turned metabolic labeling with heavy water coupled with LC–MS into a high-throughput method to analyze protein turnover [4,14]. Bioinformatics tools [8,15,16,17] have been developed to automate the data analysis from high-resolution mass spectrometry (HRMS). The metabolic labeling with heavy water is cost-effective compared to labeling with other stable isotope precursors (e.g., labeling with heavy amino acids, such as Lys [18]). It labels all non-essential amino acids, lipids [19,20], nucleic acids [21], and other biomolecules [22]. The proteome content of tissues is analyzed in LC–MS using techniques of bottom-up proteomics [23]. Database search engines are used to identify peptides/proteins [24,25]. The isotope profiles of identified peptides are quantified in MS^1^ (survey scans). The incorporation of the deuterium leads to the changes in the distribution of the mass isotopomers [26]. Bioinformatics tools model the changes with exponential time-course functions to extract degradation rate constants [27,28,29].

The development of bioinformatics tools has automated the protein rate constant estimations. However, it is estimated that only about 30–45% of all quantified peptides are used in the rate constant estimations [15,30]. While heavy water labels all non-essential amino acids and virtually all peptides, some peptides do not pass the goodness-of-fit (such as the correlation between theoretical fit and experimental data, residual sum of squares) filtering. Furthermore, some proteins have a high coefficient of variation as calculated from the rate constants of constituent peptides. As in other quantification approaches, one contributing factor is distortion of the mass isotopomer distributions of target peptides by the co-eluting contaminants. A potential strategy to overcome this problem is to increase mass resolution in data collection of survey scans (MS^1^ scans). HRMS mass analyzers allow the separations of co-eluting species with close mass-to-charge ratio (*m*/*z*). The high mass resolution (R) provides the ability to exclude distortions of the isotopic profiles of target species by co-eluting contaminants. However, quantification of label (deuterium) incorporation also relies on accurate measurements of the relative abundances (RAs) of the mass isotopomers. The improvements in spectral accuracy (accuracy of the relative abundances of the mass isotopomers [31]) of the modern mass analyzers have lacked compared to those of the mass measurements [32].

Recently, murine blood serum protein turnover has been analyzed at various settings of the mass resolution in the Fusion Orbitrap mass analyzer [16]. Several settings of the resolution have been used. It was observed that the best performance was obtained at 60,000 resolution [16]. The samples were also analyzed with the Agilent QToF mass analyzer with 10,000 resolution. The number of quantified proteins and the rate constant estimation worsened when the mass resolution was set at 120,000 or higher. Here we analyze the data set with d2ome [8], the bioinformatics tool we have developed for protein turnover studies. We evaluate the dependence of rate constant estimations on the Orbitrap mass analyzer’s mass resolutions. D2ome computes the RAs of mass isotopomers from profiled data (in MS^1^). In d2ome, the mass isotopomer abundances are computed in three-dimensional space of *m*/*z*, time, and abundance. It also calculates the peak widths of mass species, which we compare for the different mass resolutions. For various settings of the mass resolution, estimates of spectral accuracy are obtained by comparing the errors in the RA of the monoisotope for non-labeled samples.

## 2. Results and Discussions

**Figure 1:**
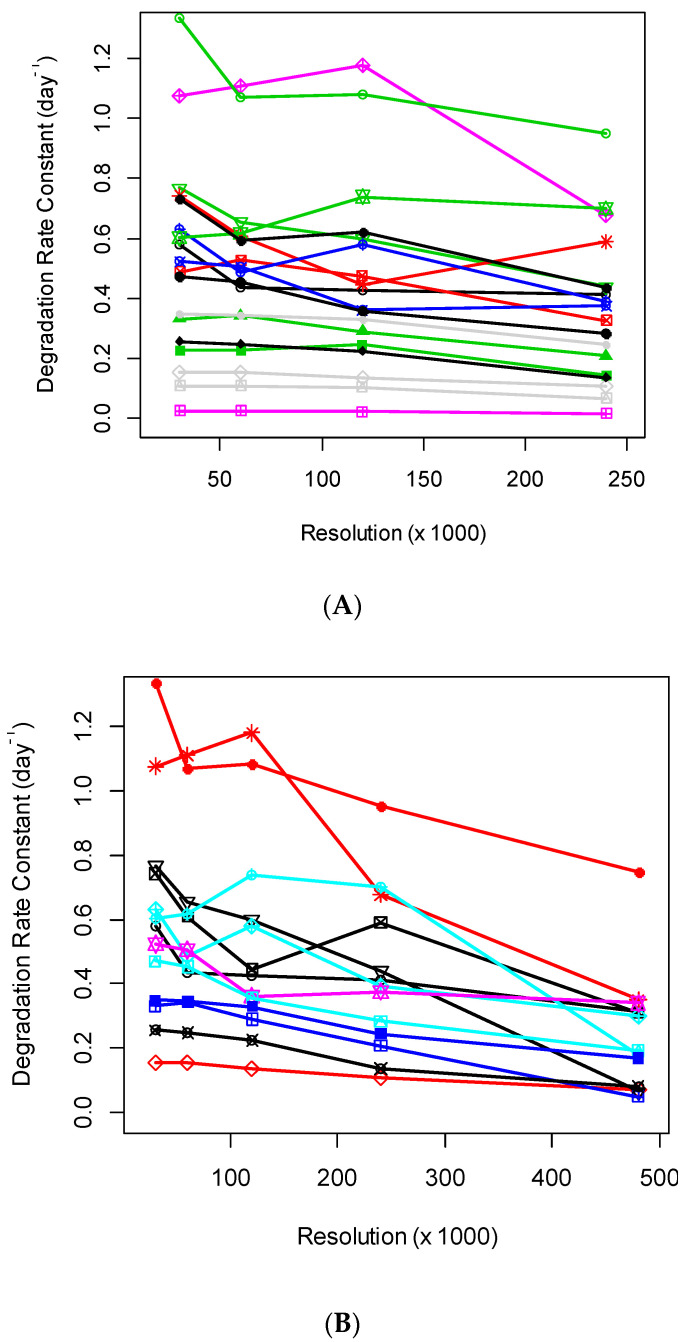
(**A**) Degradation rate constants, which were computed using isotope profiles recorded in the Orbitrap mass analyzer, were relatively stable for mass resolutions up to 120,000. Degradation rate constants were at four different MS^1^ level resolutions. Shown are the data for 18 blood serum proteins quantified by at least three peptides in every experiment. The degradation rate constants were produced by d2ome. (**B**) Degradation rate constants of proteins/peptides quantified in Orbitrap mass analyzer were reduced at the mass resolution of 480,000. Degradation rate constants were at five different MS^1^ level resolutions. Shown are the data for thirteen blood serum proteins quantified by at least three peptides in every experiment. The degradation rate constants were obtained using d2ome. Each combination of a symbol and color represent the rate constant of a protein at specific resolution.

The increase in MS resolution allows the separations of profiles of co-eluting species. The separations reduce the interferences from contaminations and may improve the quantification (assuming that the spectral accuracy does not deteriorate). In practice, we observed that the increased mass resolution in the Orbitrap mass analyzer resulted in a reduced number of identified and quantified peptides and adversely affected the rate constant estimations. The number of proteins, for which rate constants were possible to estimate at the various settings of the mass resolution in the Orbitrap mass analyzer, is shown in Table 1. As seen from the table, the number of quantified proteins and peptides tended to reduce with the increase in mass resolution. This is in agreement with the previous observation made using DeuteRater [16]. The changes in the mass resolution affected the calculated rate constants as well, as shown in Figure 1A,B. There were 18 blood serum proteins common to the data sets with R = (30,000; 60,000; 120,000; 240,000) resolutions, identified and quantified by at least three unique peptides. Their estimated rate constants at various resolutions are shown in Figure 1A. When we include the data for R = 480,000, the number of proteins with three or more quantified peptides reduced to thirteen. As seen from the figure, the protein degradation rate constants tended to decrease when the resolution increased higher than 120,000. At 480,000 resolution, the rate constants were reduced for all but one protein. This trend was reflected in the linear regression coefficients of the rates on the resolution (see below the discussions for the ten most abundant proteins).

**Figure 2:**
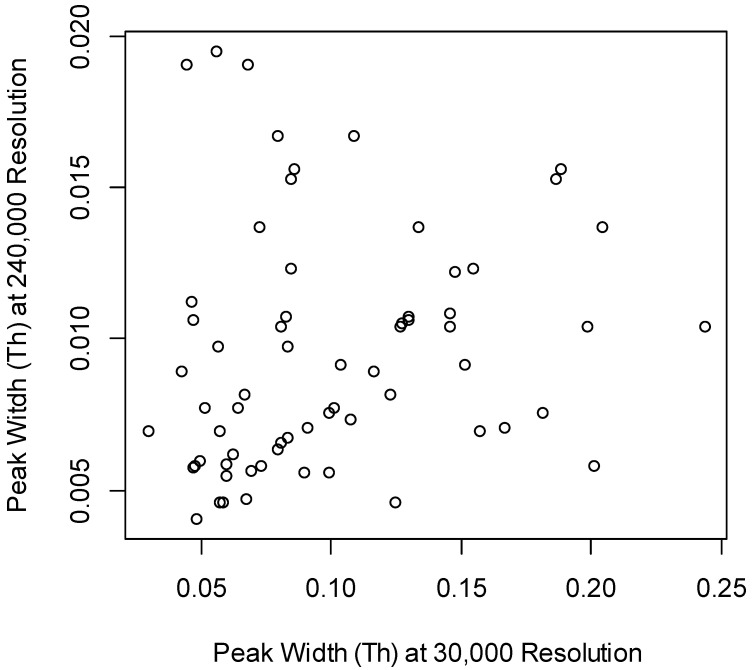
For the increased resolution, the peak widths were smaller. The scatter plot of peak width computed by d2ome for the serum Albumin peptides quantified at 30,000 (x-axis) and 240,000 (y-axis) resolutions. The ratio of the means of the peak widths was 10.5.

d2ome uses profile data (in MS^1^) for estimating mass isotopomer profiles of peptides. It determines the width of the monoisotopic peaks in the *m*/*z* domain, before estimating isotope abundance. Figure 2 shows the scatter plot of the widths of monoisotopic peaks of Albumin (the most abundant protein by spectral count and by total ion current of its peptides) peptides computed at 30,000 and 480,000 MS resolutions. On average, the peak widths at 30,000 MS resolution were ten times larger than the corresponding values at 480,000 MS resolution. d2ome detected the increased mass resolution (that resulted in the narrow peak widths). This result confirmed that the increased resolution resulted in the narrowing of isotope peak profiles in MS^1^. The narrowing of the peaks reduces the chances of interferences from the co-eluting contaminants. However, as can be seen below, the increased resolution also affected the spectral accuracy of isotope profiles and reduced the number of identified and quantified peptides.

**Figure 3:**
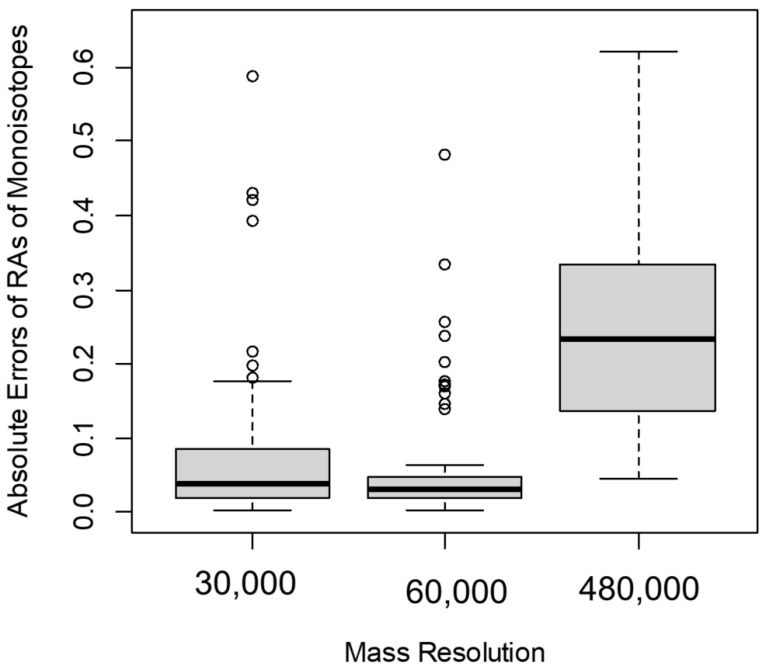
In the Orbitrap mass analyzer, the accuracy of the measurements of RAs of monoisotopes was affected by the mass resolution. The boxplot of the errors (defined in the main text) of the RA monoisotopic peaks, *I*_0_(0), at 30,000, 60,000, and 480,000 MS^1^ resolutions.

To evaluate the spectral accuracy, we used the natural (non-labeled) isotope profiles of peptides at various mass resolutions. We compared the differences between the estimated values of RA of monoisotopic peaks and the corresponding theoretical values (for non-labeled peptides) at different mass resolutions. Figure 3 shows the boxplots of the errors in the estimation of the RA of the monoisotope *I*_0_ before labeling at three different mass resolutions: 30,000, 60,000, and 480,000. We defined the error as the ratio of the absolute value of the difference between the experimental value $I_{0}^{expr}\left(0\right)$ and the theoretical value $I_{0}^{theor}\left(0\right)$ to the theoretical value (1)

(1) $$Error=abs\left(I_{0}^{theor}\left(0\right)-I_{0}^{expr}\left(0\right)\right)/I_{0}^{theor}\left(0\right)\;$$

When the MS resolution increased from 30,000 to 60,000, the errors for 75% of the Albumin peptides decreased (Figure 3). In contrast, at the MS resolution of 480,000 for more than 95% of serum Albumin peptides, the error in the estimation of *I*_0_(0) increased compared to the corresponding values from 60,000 MS resolution (Figure 3). We obtained the significance of the observed errors from different mass resolutions using t-tests. The t-statistic between the errors from 60,000 and 30,000 resolutions was relatively high and equal to −1.7. The corresponding *p*-value, 0.08, was small, but it was not statistically significant. The 95% confidence interval of the test was [−0.1, 0.01]. As is seen in the boxplot (Figure 3), medians of the errors were close. However, the interquartile range was smaller for the errors observed from the data obtained at 60,000 resolution.

**Figure 4:**
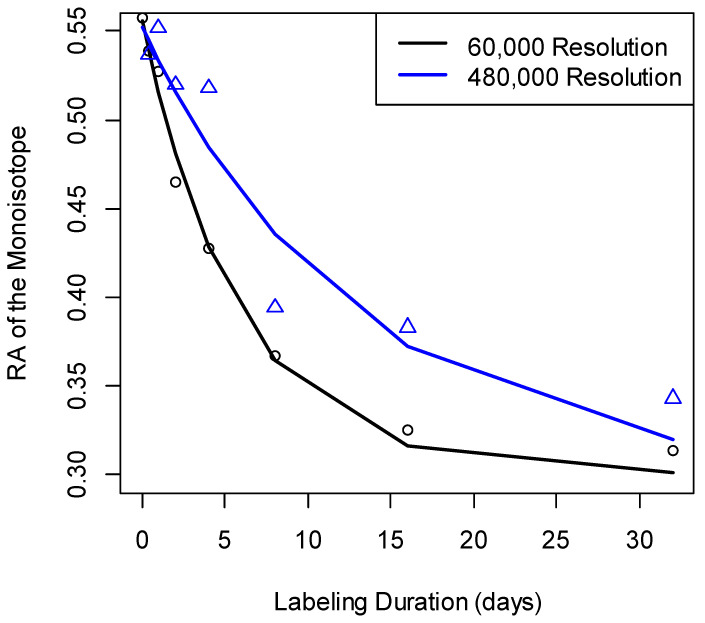
The changes in RA estimation that accompanied the increase in mass resolution affected the rate constant estimation. Measurements (empty circles, and triangles) of the RA of the monoisotopic peak for the Albumin peptide “TNCDLYEK” and their fits (lines). The fit to the experimental data at 60,000 resolution (black line) produced the rate constant of 0.1724 day^−1^. The rate constant from the data at 480,000 resolution (blue line) was 0.077 day^−1^.

For the comparison between the errors at 60,000 and 480,000 resolutions, the t-statistic was equal to −7.7. This statistic was highly significant (the *p*-value was in the order of 10^−11^). The 95% confidence interval of the test was [−0.24, −0.14]. The median of errors at 480,000 MS^1^ resolution was higher than those at the other two resolutions (Figure 3). Overall, for the mass resolutions used in the data sets of this study, the best accuracy of *I*_0_(0) estimation was achieved at 60,000 mass resolution. In addition, 80% of Albumin peptides had better accuracy at R = 60,000 than at R = 120,000. The reduced spectral accuracy affected the time-course data of peptides. Figure 4 shows the time course of the depletion of the RA of the monoisotope as computed from spectral data at two different mass resolutions, 60,000 and 480,000, for Albumin peptide “TNCDLYEK”. The increased resolution resulted in the overestimation of the RA of the monoisotope and led to reduced rate constant estimation.

Table 2 presents the values of the rate constants for the ten abundant proteins in the murine blood serum samples. We compared the values obtained from various mass resolution settings of the Orbitrap mass analyzer with the corresponding results we obtained using mass spectral data of the QToF. It has been reported [33,34] that the QToF mass analyzers provide better spectral accuracy compared to the Orbitrap mass analyzers. As seen in Table 2, for the abundant proteins, d2ome produced results consistent with those from QToF data analysis at the resolutions up to 120,000. The exceptions were the proteins Carboxylesterase 1C (gene name, EST1C_MOUSE) and Complement C3 (gene name, CO3_MOUSE) in the QToF replicate experiment. The rate constants for these proteins obtained in the second replicate from QToF data were different from the first experiment and from the corresponding values obtained in the data of the Orbtrap mass analyzer (at 30,000 and 60,000 mass resolutions). The rate constants showed consistent decrease as the resolution of the Orbitrap mass analyzer increased. Additionally shown in the table are the coefficients of the linear regressions of protein rate constants on the mass resolution for each protein. All regression coefficients were negative. The coefficients were statistically significant for most of the rate constants.

**Figure 5:**
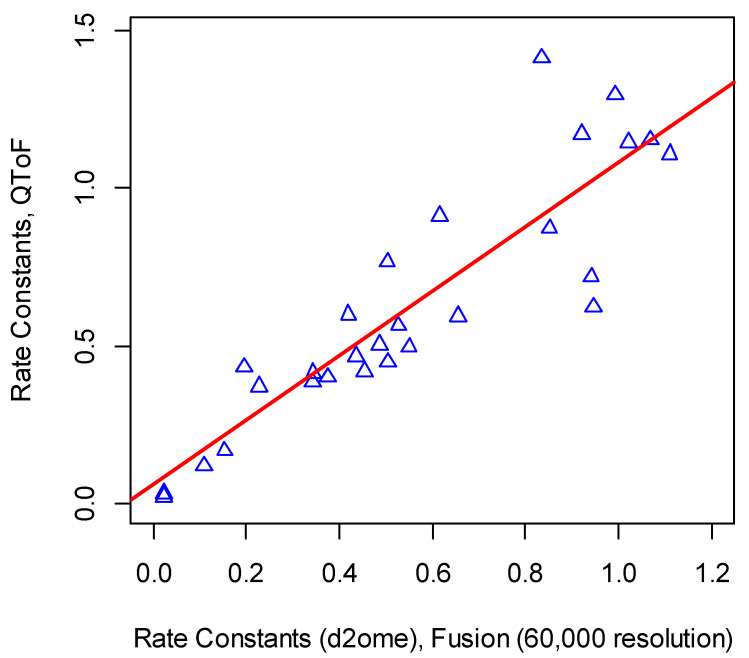
The proteome dynamics from QToF 10,000 resolution and Orbitrap 60,000 resolution were comparable. The scatter plot of the rate constants from QToF and Orbitrap Fusion.

For a more detailed comparison with QToF data, we first determined the quantified proteins in replicate QToF experiments. The Pearson correlation between the rate constants of proteins from QToF and Orbitrap samples was 0.89. The slope of the linear regression of QToF rate constants on the d2ome rate constants using the Orbitrap (R = 60,000) data was 1.1, with *p*-value in the order of 10^−16^. The intercept was 0.05, and its *p*-value was insignificant and equal to 0.38. In Figure 5, we show the scatter plot of the rate constants and the linear regression line.

**Figure 6:**
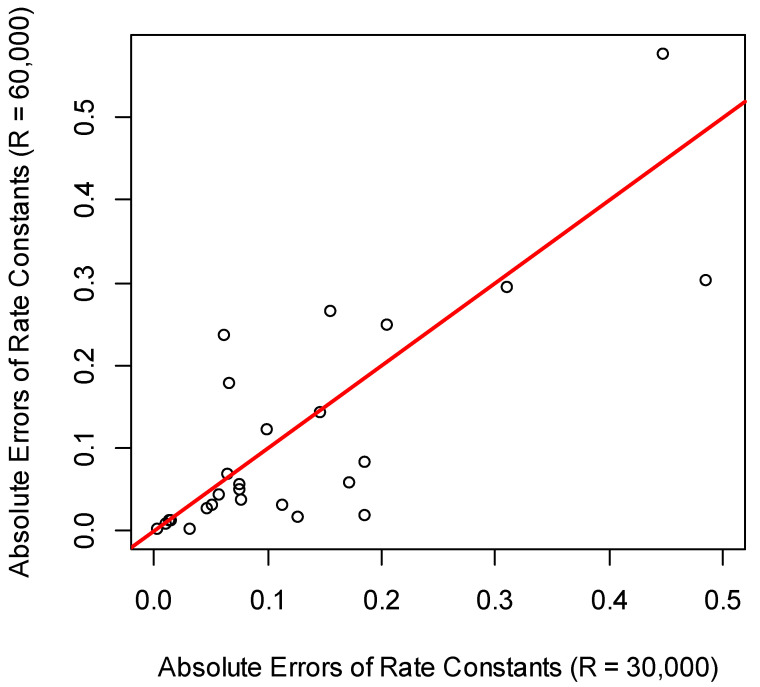
The scatter plot of the absolute values of the differences between each setting of the resolution (R = 30,000 and 60,000) and the corresponding rate constants from the QToF data.

The number of quantified proteins was the highest in the experiment using 30,000 resolution (Table 1). For comparing the rate constants produced from data at R = 30,000 and R = 60,000, we used the rate constants from QToF data. The scatter plot of the absolute values of the rate constants’ differences at each resolution and the QToF result are shown in Figure 6. Both the linear regression and correlation between the rate constants of R = 60,000 and QToF were better than those between R = 30,000 and QToF. For R = 30,000, the slope and intercept of the linear regression were 0.5 and 0.2, respectively. Both values were statistically significant. For 67% of rate constants, the absolute errors from R = 30,000 resolution were higher than those from R = 60,000 resolution. Combining this information and the values of the slope of the linear regression (1.01 vs. 0.5) suggests that R = 60,000 resolution produced better results for rate constant estimations (when compared to QToF data as standard).

While the increase in mass resolution can potentially improve the rate constant estimations, in practical applications, the best rate constant estimations (using QToF results as standard) were obtained for R = 60,000. We showed that the decreased accuracy of the rate constant estimation was related to the reduced spectral accuracy observed at high resolutions (Figure 1A,B). We note that the data set for this analysis was not large-scale (less than 100 proteins were analyzed). In complex samples, the contaminants’ effects on the mass isotopomer profiles of the target peptides are expected to be higher. The increased mass resolution may help to separate the co-eluting species. However, the reduced mass spectral accuracy will still hamper the accurate rate constant estimations at higher resolutions of the Orbitrap mass analyzer.

## 3. Materials and Methods

**Figure 7:**
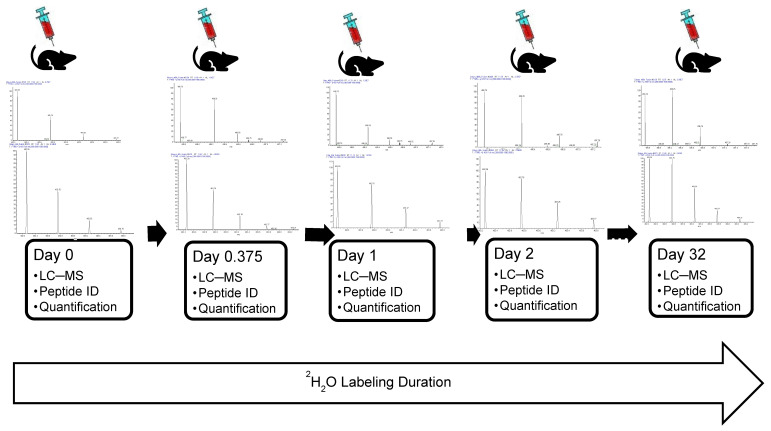
A schematic of the metabolic labeling and LC–MS experiments for estimating protein turnover. Blood is drawn from the mouse after certain intervals of labeling with heavy water. Shown are isotope profiles of the peptide “VDGALCLDK” of Hemopexin. The three periods indicate additional time points of label duration. The isotope distributions are those at the apex of the elution profile in LC. The isotope distributions were obtained with resolutions of 480,000 (upper panel) and 60,000 (lower panel).

We used a publicly available data set [16] from a proteome dynamics study of murine blood serum proteome acquired on the Orbitrap Fusion (ThermoFisher Scientific, San Jose, CA, USA) mass spectrometer. d2ome quantifies stable isotope label incorporation at the MS^1^ (full scan) level. The mass spectral data were obtained at five different settings of the resolution at MS^1^ level: 30,000, 60,000, 120,000, 240,000 and 480,000. The blood samples were taken from a mouse (strain C57) at eight different time points of label exposure: 0, 0.375, 1, 2, 4, 8, 16, and 32 days. Peptide sequences were identified from the tandem mass spectra. The fragment ions resulting from the collision-induced dissociation of intact peptides were recorded in linear ion trap. Figure 7 depicts the schematic of the experiments. 

The temporal dynamics of deuterium incorporation in murine blood serum proteome was also analyzed with Agilent 6530 QToF (Agilent Technologies, Inc., Santa Clara, CA, USA) at 10,000 *m*/*z* resolution. For the QToF data, tandem mass spectra were collected only on unlabeled peptides. It was reported that this approach (longer dwell times in MS^1^ acquisition of labeled samples) allowed better signal-to-noise for the measurements of mass isotopomer abundance [16]. The QToF data were suggested as a standard for comparison [16]. Spectral accuracy [31] (accurate measurements of relative abundances of mass isotopomers) of the QToF mass analyzers was higher than those of the Orbitraps [33]. The data sets are not large-scale. They contain only a few dozen proteins. However, they were obtained from the same mice, and peptide samples were identical. Therefore, they are appropriate for rate constant calculations at various settings of the mass resolution.

We used the Mascot [25] database search engine for peptide/protein identifications. The values database search parameters were as follows: precursor mass accuracy was set to 15 ppm; fragment ion mass accuracy was set to 0.6 Da (Orbitrap Fusion) or 40 ppm (QToF); carbamidomethylation of Cys was the fixed modification; oxidation of Met and acetylation of Lys were set as dynamic modifications; the number of ^13^C was set equal to two. Trypsin specificity with up to two missed cleavages was used. The Swiss-Prot database (downloaded in May 2017) and mouse taxonomy were used. The global false discovery rate (FDR) was controlled using the decoy database approach. The FDR was set at 1%.

All mass spectral data sets and the database search results are freely available. The relevant references can be found in the original publication [16]. The data specific to this study are provided in the Supplementary Materials.

### Estimation of the Rate Constant

The gradual incorporation of the label (^2^H) into a peptide occurs according to the labeling duration, the number of exchangeable hydrogens (N_EH_), and the degradation rate constant (proteins are assumed to be at steady state) of a protein (from which the peptide is derived). Bioinformatics tools use the time-course data of depletion of the RA of monoisotope of a peptide to estimate the degradation rate constant. The time course of the depletion of the RA of the monoisotopic peak $I_{0}\left(t\right)$ is modeled as (2):

(2) $$I_{0}\left(t,\;k,\;N_{EH}\right)=I_{0}^{asymp}+\left(I_{0}\left(0\right)-\;I_{0}^{asympt}\right)*e^{-kt}$$

where $I_{0}\left(0\right)$ is the RA of the monoisotope before the start of the labeling (RA of the monoisotopic peak from the naturally occurring isotopes), *I*_0_^*asymp*^ is the asymptotical (after reaching labeling plateau) RA of the monoisotope, and k is the peptide/protein degradation rate constant (estimated via fit to the experimental data). *I*_0_^*asymp*^ is determined from the body water enrichment (BWE) of the deuterium *p* and the *N_EH_* of a peptide via the Equation (3) [14,15,35]:

(3) $$I_{0}^{asympt}=I_{0}\left(0\right){\left(1-p/p_{0}\right)}^{N_{EH}}$$

In Equation (3), *p*_0_ is the relative abundance of ^1^H in nature. The BWE is normally estimated from the enrichments of free amino acids in the blood serum [36]. To obtain the rate constant k, an experimental set of monoisotopic RAs ${\left\{I_{0}\left(t_{n}\right)\right\}}_{n=1}^{T}$ is fit to the formula in Equation (2). Here, T is the number of experimentally sampled time points. The optimization is done via non-linear regression using the Broyden–Fletcher–Goldfarb–Shanno (BFGS) [37] algorithm to minimize the residual sum of squares.

For every peptide, d2ome computed its degradation rate constant using Equations (2) and (3). The rate constants from all peptides of a protein were analyzed for outliers using Grubbs’ outlier method. The median of the remaining rate constant was assigned to the protein.

## 4. Conclusions

We studied the estimation of protein turnover at various MS resolutions of a popular mass analyzer, the Orbitrap. The increase in mass resolution narrowed peak widths in *m*/*z* domain, which separated co-eluting species with close *m*/*z* values. d2ome, which uses profile MS^1^ data, reported reduced peak width in *m*/*z* domain with the increased MS resolution. However, the accuracy of the rate constant estimation decreased significantly with the increased MS resolutions (compared to 60,000 resolution). Data showed that the reduction was due to the deterioration in spectral accuracy.

## Figures and Tables

**Table 1:** The number of analyzed proteins at different mass resolutions of the Orbitrap Fusion.

Number of	Resolution (×1000)				
	30	60	120	240	480
quantified proteins	91	97	92	86	58
proteins with 3 or more unique peptides	32	29	23	21	13
proteins with CV ≤ 0.3	45	41	39	26	10
peptides usable for proteome dynamics	454	472	421	363	219
identified but unquantifiable proteins	13	10	16	17	35

**Table 2:** Rate constants (day^−1^) of the ten abundant proteins in the murine serum blood samples [16] computed using d2ome. The same samples were analyzed at different values of mass resolution. Additionally shown is the coefficient of the linear regression on the mass resolution for each protein.

Proteins	QToF1	QToF2	Resolution (×1000)					Linear Coefficient (×10^−^^3^)
			30	60	120	240	480	
Albumin	0.170	0.224	0.158	0.154	0.134	0.101	0.072	−0.2 **
Serotransferrin	0.391	0.455	0.340	0.344	0.329	0.244	0.168	−0.4 **
Alpha-2-macroglobulin	0.309	0.352	0.258	0.260	0.235	0.136	0.080	−0.4 **
Hemopexin	0.387	0.504	0.573	0.505	0.360	0.375	0.339	−0.4
Apolipoprotein A-I	0.629	0.570	0.768	0.654	0.599	0.438	0.069	−1.5 **
Complement C3	1.277	2.08	1.019	1.174	1.169	0.788	0.142	−2.2 *
Carboxylesterase 1C	0.522	0.215	0.625	0.414	0.538	0.392	0.300	−0.5
Murinoglobulin-1	0.321	0.374	0.343	0.316	0.247	0.161	−0.031	−0.7 **
Serine protease inhibitor A3K	0.466	0.497	0.466	0.453	0.412	0.284	0.193	−0.6 **
Transthyretin	1.087	1.203	1.095	1.02	0.877	0.95	0.620	−0.9 *

* the *p*-value of the linear coefficient is significant (less than 0.05). ** the *p*-value of the linear coefficient is highly significant (less than 0.01).

## Supplementary Materials

Supplementary materials can be found at https://www.mdpi.com/1422-0067/21/21/7821/s1. The table of protein rate constants, the standard deviations of the rate constants, spectral count, and total ion current for proteins quantified at various mass resolutions.

## Abbreviations

BWE	body water enrichment
HRMS	high-resolution mass spectrometry
LC–MS	liquid chromatography and mass spectrometry
m/z	mass-to-charge ration
NEH	number of exchangeable hydrogens
QToF	quadrupole time-of-flight
R	resolution
RA	relative abundance
